# New animal models of neurocysticercosis can help understand epileptogenesis in neuroinfection

**DOI:** 10.3389/fnmol.2022.1039083

**Published:** 2022-11-16

**Authors:** Hector H. Garcia, Manuela R. Verastegui, Gianfranco Arroyo, Javier A. Bustos, Robert H. Gilman, for The Cysticercosis Working Group in Peru

**Affiliations:** ^1^Center for Global Health, Universidad Peruana Cayetano Heredia, Lima, Peru; ^2^Cysticercosis Unit, Instituto Nacional de Ciencias Neurologicas, Lima, Peru; ^3^Asociación Benéfica PRISMA, Lima, Peru; ^4^Alberto Cazorla School of Sciences and Philosophy, Universidad Peruana Cayetano Heredia, Lima, Peru; ^5^Department of International Health, Johns Hopkins Bloomberg School of Public Health, Baltimore, MD, United States

**Keywords:** *Taenia solium*, larval cestodes, cysticercosis, neurocysticercosis, epilepsy, Peru

Neurocysticercosis (NCC), defined as an infection of the central nervous system (CNS) by the cystic larval stage of the pork tapeworm *Taenia solium*, remains a major challenge in public health mainly due to associated neurological morbidity (Garcia et al., 2020; Bustos J. et al., 2021). This infection is endemic in most of the developing world including Central and South America, Sub-Saharan Africa, and large regions of Asia including India, China, and Southeast Asia (Ndimubanzi et al., 2010; Garcia et al., 2020; Bustos J. et al., 2021). NCC is also increasingly diagnosed in non-endemic countries and industrialized countries due to immigration and travel from endemic zones (O'Neal et al., 2011; Gabriël et al., 2015; O'Neal and Flecker, 2015).

*T. solium* has a complex two-host life cycle. Humans acquire intestinal taeniasis by ingesting poorly cooked pork containing the parasitic cystic larvae or cysticerci. Once the adult tapeworm develops in the human small intestine, its microscopic eggs are shed with the stools. In places with poor sanitation and domestic pig raising, pigs ingest contaminated human feces containing *T. solium* eggs. Ingested *Taenia* eggs release their embryos, called oncospheres, which cross the intestinal mucosa, and are distributed by the circulatory system throughout the body. They evolve into metacestodes (post-oncospheral stage) and encyst to form larval vesicles or cysticerci, reaching their definitive size in 2–3 months. These infected pigs become the intermediate host by being infected with the larval stage of the infection (cysticercosis), and thus the source of taeniasis in the community. Humans can also act as intermediate host instead of pigs and become infected with cysticercosis through accidental ingestion of *Taenia* eggs by fecal oral infection from a tapeworm carrier in their surroundings (Flisser, 1994).

Clinical symptoms of NCC predominantly result from involvement of the CNS. Outside the nervous system, cysticercosis causes few or no symptoms and the cysts are usually identified and destroyed by the host's immune response. The clinical manifestations, diagnostic and therapeutic approaches, and prognosis of NCC vary enormously depending on the type, stage, location, number and size of parasites in the nervous system, as well as the immune response of the host (Garcia et al., 2020; Bustos J. et al., 2021). The most frequent clinical manifestations of NCC are seizures, headaches, and intracranial hypertension. In endemic regions, NCC is considered the most frequent cause of acquired seizures and epilepsy, accounting for up to 30% of seizure disorders in these areas (Ndimubanzi et al., 2010; Mazumder and Lee, 2022; Segala et al., 2022; Takayanagui and Haes, 2022).

Once in the brain parenchyma, the larvae establishes as a viable cyst, protected by the blood brain barrier, and enacts a series of immune evasion mechanisms that enable it to survive for long periods, that may easily reach several years or even more than a decade. During this time, there is only a mild inflammatory reaction around the cyst. At some point and by reasons not yet known, the host's immune system detects the parasite and attacks it with a cellular response, release of pro-inflammatory cytokines, and marked local inflammation that leads to the death of the parasite and its collapse of the cyst into an inflammatory nodule. Eventually, the inflammatory process subsides and most of the cyst remains are reabsorbed (Gonzales et al., 2016). In a significant proportion of cases (~20–30% in single lesion NCC, 40% in multilesional NCC) the lesions end in a well-defined calcified scar easily seen on computed tomography (Bustos J. A. et al., 2021). There is little doubt, if any, that NCC causes seizures and epilepsy. Seizures are less frequent in individuals with only viable cysts, highly frequent in cases with degenerating cysts, and persist in 50% of more of patients after the cysts have resolved, with a strong association between the likelihood of seizure relapses and the presence of a calcified scar. Frequently patients with NCC present to the neurologist only after years with seizures, usually of the same type and topographically related to their parasitic lesions (Garcia and Del Brutto, 2017; Duque et al., 2018). Seizures are usually well-controlled with first line AEDs but may be refractory in a small proportion of cases. Surgical series of temporal lobe epilepsy have found a significant association between the presence of calcified NCC lesions away from the hippocampus and hippocampal sclerosis, suggesting that NCC may not only act as an epileptogenic lesion causing local damage in the case of temporal cysts, but also as an initial precipitating injury to cause hippocampal sclerosis years later. Even if located distant from the hippocampus. Whether this is due to subclinical seizure activity or inflammatory mediators is not yet known (Singh et al., 2013; Del Brutto et al., 2021; Secchi et al., 2022).

The study of brain damage and epilepsy in human NCC has always been hampered by the lack of reliable animal models. Controlled animal models can greatly help to understand the processes associated with NCC infection, study the host-parasite interactions, ascertain the immunopathological and inflammatory processes associated with CNS cyst infection and in response to antiparasitic treatment, define biomarkers for human disease, and also provide an ideal scenario for testing novel therapies in controlled studies (Arora et al., 2017; De Lange et al., 2019).

Rodent NCC models offer multiple advantages for the study of NCC, such as the ability to include large numbers of animals in experiments, availability of commercial reagents for analysis, and comparability with studies in other diseases (Arora et al., 2017; De Lange et al., 2019; Sitali et al., 2022). Intracranial injection of *Mesocestoides corti* or *Taenia crassiceps* in mice has been used to characterize the processes of neuroinflammation and cerebral granuloma formation (Patil et al., 2006; Alvarez et al., 2010). These models, although successful to produce CNS infection, are limited by the fact that natural CNS infection in these species is rare, and that these cysts are proliferating in nature, unlike *T. solium* NCC. Therefore, *T. solium* animal models are needed to understand the pathogenesis of human NCC.

Experimental *T. solium* infections in mice were described as early as in 1994 (Yang et al., 1994) and 1997 (Ito et al., 1997) by intravenous or subcutaneous injections. Better results are obtained with immunosuppressed mice, in which 40–76% develop cysticercosis through intravenous infection, and 100% when infected subcutaneously. No central nervous system cysts were obtained in these studies (Ito et al., 1997; Wang et al., 1999; Liu et al., 2002). Our group has successfully infected rats orally with oncospheres and produced typical CNS cysticercosis infections, without the need for immunosuppression. This method however had the disadvantage of requiring large numbers of activated oncospheres. Non immunosuppressed rats given an oral dose of 20,000 activated oncospheres produced cysts in the brain in only 17% of the infected rats (Mejia Maza et al., 2019).

More recently our group described a model of intracranial injection of activated *T. solium* oncospheres in neonate rats (Verastegui et al., 2015; Carmen-Orozco et al., 2019, 2021; Mejia Maza et al., 2019). This model is consistently efficient (more than 80% of infected rats develop viable CNS cysts, using as few as 120 activated oncospheres per rat; Carmen-Orozco et al., 2021). Although intracranial injection bypasses the intestinal mucosa step that occurs in natural infection and oral infection models, cysticerci found in the rat brains (mainly 1–2 cysticerci per brain) show similar characteristics to those found in oral rat infections (Sitali et al., 2022) or in the natural hosts (pigs and humans), making this model more appropriate to study NCC compared to previous rodent models (Verastegui et al., 2015; Mejia Maza et al., 2019).

In the rat model our group has demonstrated similar pathology of the brain surrounding the cysts to that occurring in human neurocysticercosis. Cerebral *T. solium* cysts in the rat are associated with blood-brain-barrier (BBB) disruption, angiogenesis, astrogliosis, activated microglia, and axonal spheroids, all changes present in the human neurocysticercosis (Mejia Maza et al., 2019). Dispersion of *T. solium* antigens and increased gene expression of cerebral pro-inflammatory cytokines around brain cysticerci, and distant from cysticerci have also been demonstrated (Verastegui et al., 2015; Carmen-Orozco et al., 2019, 2021; Mejia Maza et al., 2019). Moreover, an increase in dysfunctional autophagy has been observed in the neurons surrounding cysticerci in the rat model, which also colocalizes with axonal swellings and supports the hypothesis that dysfunctional autophagy is involved in the pathogenesis of NCC. A subgroup (~9%) of intracranially infected rats develop evident generalized tonic-clonic seizures by 4 months after NCC infection (Verastegui et al., 2015), and this proportion increases to 40% by 8 months post infection (*M. Verastegui, personal communication, 2022*). Epilepsy has only been shown before in an experimental model of Rhesus monkeys (Chowdhury et al., 2014), and pigs with NCC (Christensen et al., 2016; Trevisan et al., 2016), but not in rodents. In both cases seizures were seen in animals with heavy brain infections. Chowdhury et al. (2014) infected Rhesus monkeys with 6,000 or 12,000 infective eggs and achieved heavy brain infections, with very severe symptoms as early as 10 days post infection. Trevisan et al. (2016) observed and videotaped 16 pigs with NCC and uninfected controls and found clinically apparent seizures in two. Large collagen fibrotic scars were found in the two pigs with seizures but not in controls (Christensen et al., 2016).

This novel rat model of NCC allows the correlation of histopathological findings with seizure activity by using telemetric encephalogram with a small set of electrodes in the rat skull, so that EEG recordings can be later correlated with focal histopathological findings as in other rat epilepsy models. The anatomical pattern of brain infection includes intraparenchymal cysts in a large majority of animals (some with diverse degrees of contact with the subarachnoid space), with occasional intraventricular cysts, as in humans. It does not preferentially follow a particular vascular territory (Verastegui et al., 2015; Carmen-Orozco et al., 2019, 2021; Mejia Maza et al., 2019). However, a main drawback of this and other rodent NCC models is the large size of the cyst (~5 mm) in relation to the rat brain or skull, that may cause mass effects and contribute to exacerbate inflammation and damage associated with cyst establishment, limiting its use in translational studies. Clearly, not all the pathology in this model is related to mass effects or compression, since axonal swelling is present in all directions around the parasite and not increased in areas next to the skull where compression would have been higher (Mejia Maza et al., 2019).

The pig, on the other hand, is considered the best model for the study of NCC. Pigs are the natural hosts of the *T. solium* larvae, and the immunopathological characteristics of CNS cyst infection in pigs closely resemble the human pathology (De Lange et al., 2019; Sitali et al., 2022). Studies in naturally infected pigs have been previously reported to assess the efficacy of cysticidal drugs (Gonzalez et al., 1995, 1997, 1998, 2001, 2012; Gonzales et al., 1996; Vargas-Calla et al., 2016), immunotherapies (Evans et al., 1997; Verastegui et al., 2003), and diagnostic test performance (Gonzalez et al., 1990; Bustos et al., 2019). Of particular interest, studies in pigs naturally infected with NCC have assessed the characteristics of the perilesional, acute brain inflammation that is triggered by antiparasitic treatment (Guerra-Giraldez et al., 2013; Marzal et al., 2014; Cangalaya et al., 2015, 2016; Mahanty et al., 2015). BBB disruption in the brain tissue adjacent to cysticerci is evident in MRI and histology as early as 2 days post-treatment and more marked by day 5 post-treatment. Neuroinflammation is found around cysts that show BBB disruption (macroscopically demonstrated as Evans Blue extravasation), and its severity correlates with increased gene expression of pro-inflammatory cytokines (IL-6, IFN-γ, TNF-α, and IL-13; Mahanty et al., 2015). The overexpression of pro-inflammatory cytokines can also be downregulated by administration of TNF-α inhibitors such as etanercept before antiparasitic treatment (Mahanty et al., 2017). However, major drawbacks of using naturally NCC pigs in pre-clinical studies includes the uncertainty about cyst longevity (unknown date of infection), pre-existing inflammation, and the extreme variability in CNS burden among NCC pigs (Arora et al., 2017; Alroy et al., 2018).

Experimental NCC pig models using oral ingestion of *T. solium* eggs or surgical implantation of oncospheres in the CNS lack reproducibility or produce mainly degenerating cysts (Santamaria et al., 2002; Deckers et al., 2008; Fleury et al., 2015). We have also recently developed and optimized an intracarotid oncosphere injection NCC pig model. The carotid route allows to direct the passage of oncospheres into the CNS of pigs and produces viable CNS cysticerci, providing a more consistent model for NCC (Alroy et al., 2018; Arroyo et al., 2022). A minimal dose of 5000 oncospheres is sufficient to consistently reproduce NCC in >80% of pigs, with parasite loads and brain distribution similar to human NCC (Arroyo et al., 2022). As in the rat model, most cysts are intraparenchymal, some with diverse degrees of contact with the subarachnoid space, and with occasional intraventricular cysts, like in human infection. Since this model reproduces viable CNS cyst infection, it can be used to more appropriately study the pathways that lead to cyst establishment, brain inflammation and damage, cyst resolution and scarring, and also to test novel therapies in controlled experimental studies. Drawbacks of the carotid NCC model include the relative lack of reagents for pig immunohistochemistry or immunology and the paucity of neurological manifestations in the pig, and logistical issues related to handling large animals. The most likely explanation for the apparent absence of clinical manifestations in pigs is that pigs are usually sacrificed for human consumption at very early ages (9 months) and thus there is no time for the parasites to evolve into degeneration and break the state of immune equilibrium of the parasite with its natural host.

In the context of animal models used for the study of epilepsy, the rat and pig models of NCC are more comparable to models of traumatic brain injury (TBI) in the sense of an established lesional injury, rather than other models of seizure induction using chemoconvulsants or electrical stimulation (Vezzani et al., 2011; Korthas et al., 2022). In clinical patients, TBI has a known time of injury which is not known in NCC patients attending after months or years of infection. In animal models however, the timeline is well-defined (Figure 1), and unlike TBI, the nature and extension of the lesion are more predictable.

**Figure 1:**
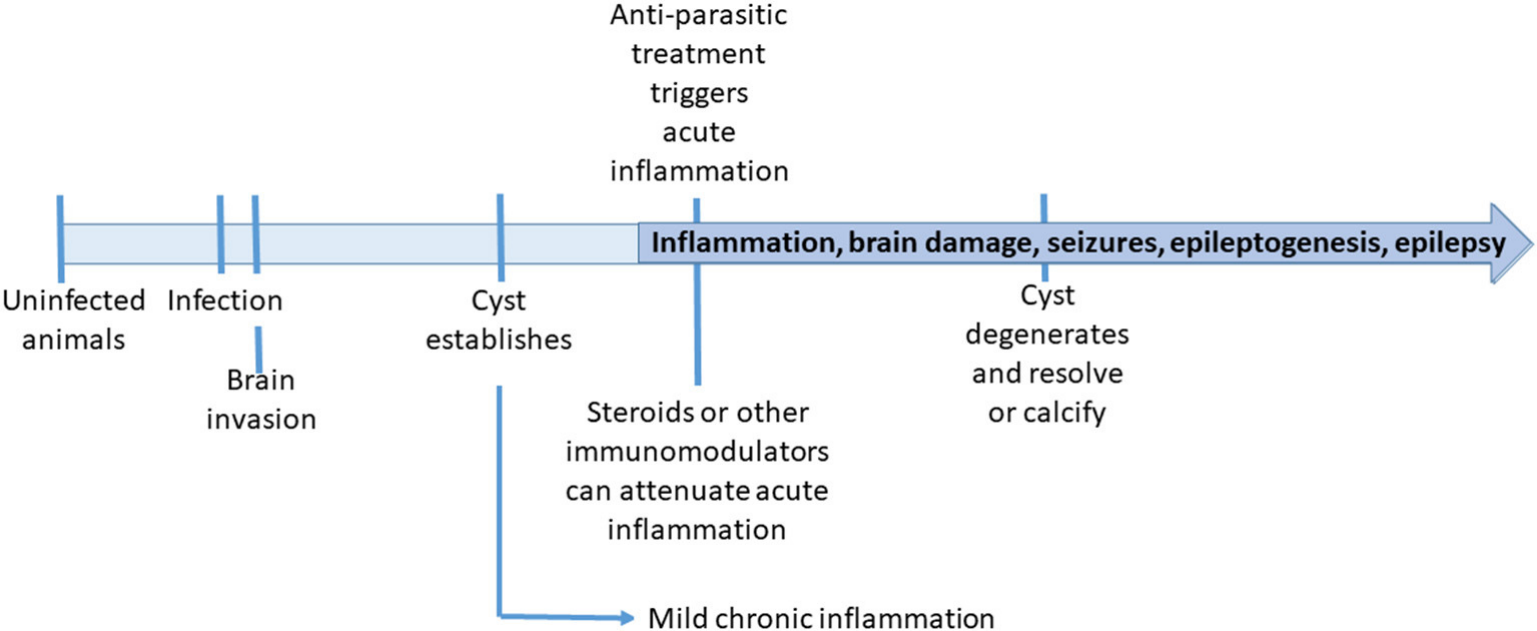
Timeline of events in the animal neurocysticercosis models.

In summary, the availability of reproducible and robust animal models for cerebral *Taenia solium* cysticercosis infection, in which the time points for lesion development and brain inflammation and damage are set, should now serve to define the mechanisms underlying brain damage and epileptogenesis that may include dysfunctional autophagy, alterations in axonal transport, and apoptosis, among others. Transcriptomic studies can contribute to elucidate the pathways related to brain inflammation and brain damage during CNS cyst infection and after antiparasitic treatment (Vezzani et al., 2019). Sound study designs involving antiparasitic and anti-inflammatory agents as well as other novel therapies that could reduce the likelihood of calcification such as bisphosphonates may lead to define the roles of inflammation and scarring as well as the interactions between these processes and how does epileptogenesis occur. Modulating inflammation, reducing damage, and decreasing residual calcification may eventually result in a much reduced likelihood of NCC-associated epilepsy.

## Author contributions

HG, MV, GA, JB, and RG contributed in the conceptualization of the manuscript, writing the manuscript, and approving the final version for submission. All authors contributed to the article and approved the submitted version.

## Funding

Work reported here was partially funded by NIAID-NIH grants U19AI129909, R01AI150544, and R01AI143553 and FIC-NIH grant D43TW001140.

## Conflict of interest

The authors declare that the research was conducted in the absence of any commercial or financial relationships that could be construed as a potential conflict of interest.

## Publisher's note

All claims expressed in this article are solely those of the authors and do not necessarily represent those of their affiliated organizations, or those of the publisher, the editors and the reviewers. Any product that may be evaluated in this article, or claim that may be made by its manufacturer, is not guaranteed or endorsed by the publisher.
